# A growing global network’s role in outbreak response: AFHSC-GEIS 2008-2009

**DOI:** 10.1186/1471-2458-11-S2-S3

**Published:** 2011-03-04

**Authors:** Matthew C Johns, Ronald L Burke, Kelly G Vest, Mark Fukuda, Julie A Pavlin, Sanjaya K Shrestha, David C Schnabel, Steven Tobias, Jeffrey A Tjaden, Joel M Montgomery, Dennis J Faix, Mark R Duffy, Michael J Cooper, Jose L Sanchez, David L Blazes, the AFHSC-GEIS Outbreak Response Writing Group

**Affiliations:** 1 Armed Forces Health Surveillance Center, 11800 Tech Rd, Silver Spring, MD 20904, USA; 2 Armed Forces Research Institute of Medical Sciences, 315/6 Rajavithi Rd., Bangkok, Thailand 10400; 3 Walter Reed/AFRIMS Research Unit Nepal, c/o U.S. Embassy, P.O. Box 295, Kathmandu, Nepal; 4 U.S. Embassy, Attn: MRU, United Nations Avenue, P.O. Box 606, Village Market 00621 Nairobi, Kenya; 5 Naval Medical Research Unit No. 2, Kompleks Pergudangan DEPKES R.I., JI. Percetakan Negara II No. 23, Jakarta, 10560, Indonesia; 6 Naval Medical Research Unit No. 3, Extension of Ramses Street, Adjacent to Abbassia Fever Hospital, Postal Code 11517, Cairo, Egypt; 7 Naval Medical Research Center Detachment-Peru, Centro Medico Naval “CMST,” Av. Venezuela CDRA 36, Callao 2, Lima, Peru; 8 Naval Health Research Center, 140 Sylvester Rd., San Diego, Calif. 92106, USA; 9 U.S. Air Force School of Aerospace Medicine, Epidemiology Consult Service, 2513 Kennedy Circle, Bldg 180, Brooks City Base, Texas 78235, USA; 10 U.S. Public Health Command (Provisional)-Public Health Region-Europe, Landstuhl, Germany

## Abstract

A cornerstone of effective disease surveillance programs comprises the early identification of infectious threats and the subsequent rapid response to prevent further spread. Effectively identifying, tracking and responding to these threats is often difficult and requires international cooperation due to the rapidity with which diseases cross national borders and spread throughout the global community as a result of travel and migration by humans and animals. From Oct.1, 2008 to Sept. 30, 2009, the United States Department of Defense’s (DoD) Armed Forces Health Surveillance Center Global Emerging Infections Surveillance and Response System (AFHSC-GEIS) identified 76 outbreaks in 53 countries. Emerging infectious disease outbreaks were identified by the global network and included a wide spectrum of support activities in collaboration with host country partners, several of which were in direct support of the World Health Organization’s (WHO) International Health Regulations (IHR) (2005). The network also supported military forces around the world affected by the novel influenza A/H1N1 pandemic of 2009. With IHR (2005) as the guiding framework for action, the AFHSC-GEIS network of international partners and overseas research laboratories continues to develop into a far-reaching system for identifying, analyzing and responding to emerging disease threats.

## Background

A central objective of disease surveillance systems is the early identification of infectious disease outbreaks to facilitate rapid implementation of effective control measures for minimizing disease transmission and morbidity. Although outbreak response has come a long way since John Snow’s investigation of cholera and the Broad Street pump in 19th century London [1], effective outbreak response continues to be challenging. Today, a particular challenge is the interconnected nature of our global society. Diseases cross international borders and present themselves in unique ways through a continuously changing landscape, making it difficult to rapidly identify, analyze and respond to disease outbreaks. Appropriate and effective monitoring of newly recognized disease clusters requires an established, standardized and well-maintained global surveillance system with a flexible framework for identifying and responding to such events.

In 1997, the U.S. Department of Defense (DoD) established the Global Emerging Infections Surveillance and Response System (GEIS) in response to the Presidential Decision Directive NSTC-7, which identified the need for more robust global disease surveillance [2]. In 2008, GEIS was integrated into the newly formed Armed Forces Health Surveillance Center [3]. As the name implies, the primary mission of AFHSC-GEIS is global disease surveillance and response. A large portion of this mission is accomplished through DoD overseas research laboratories, which were initially established within partner host countries to conduct research on infectious diseases of bilateral concern [4]. This capacity has subsequently been leveraged by AFHSC-GEIS for the purpose of disease surveillance and response.

Currently, five DoD overseas research laboratories serve in this capacity: the Armed Forces Research Institute of Medical Sciences (AFRIMS) in Bangkok, Thailand; the U.S. Army Medical Research Unit-Kenya (USAMRU-K) in Nairobi, Kenya; the U.S. Naval Medical Research Center Detachment (NMRCD) in Lima, Peru; the U.S. Naval Medical Research Unit No. 2 (NAMRU-2) in Jakarta, Indonesia; and the U.S. Naval Medical Research Unit No. 3 (NAMRU-3) in Cairo, Egypt. Additionally, the AFHSC-GEIS network includes substantial contributions from three U.S.-based research laboratories and a major regional medical center in Europe. The Naval Health Research Center (NHRC) in San Diego, California, conducts population-based surveillance among basic military trainees at eight of the 10 major training centers in the United States; disease surveillance among shipboard service members in the 2^nd^ (Atlantic), 3^rd^ (Pacific), and 7^th^ (Far East) U.S. Naval fleets; and infectious disease surveillance in six clinics and two hospitals along the United States-Mexico border [in collaboration with the U.S. Centers for Disease Control and Prevention (CDC) and the County of San Diego Health Department]. The U.S. Air Force School of Aerospace Medicine (USAFSAM) in San Antonio, Texas, which serves as the Air Force’s clinical reference laboratory and public health center, is the lead organization for the U.S. military’s installation-based, influenza sentinel surveillance program. The Walter Reed Army Institute of Research’s Division of Viral Diseases (WRAIR-DVD) in Silver Spring, Md., provides full-length genomic sequencing capability and conducts surveillance among U.S. civilians assigned to Department of State embassies overseas. Additionally, Landstuhl Regional Medical Center (LRMC) and the United States Army Public Command Health Region-Europe (PHCR-Europe) in Landstuhl, Germany, function as a regional military medical center and support surveillance for respiratory pathogens and other emerging infectious diseases (EID) within the U.S. European Command. Additionally, AFHSC-GEIS has been a member institution and contributing partner in the WHO’s Global Outbreak Alert and Response Network since 1999.

Rapid identification of outbreaks and support of timely response efforts are key components of complying with the World Health Organization’s (WHO) International Health Regulations (IHR) (2005), and are core focus areas and strategic goals of the AFHSC-GEIS network [5]. Support for these efforts, provided in response to host country requests for assistance with new or ongoing outbreaks, consists of a wide range of functions, such as field team support, epidemiology or consultative support, and laboratory diagnostic support. These efforts and collaborative exchanges strengthen relationships, build and maintain trust, and are a critical component of the long-standing relationships between the network partners and their sponsor host countries. Many of the partnerships between the United States and host country militaries (mil-mil) partnerships that have developed over the years have served to empower the host country military’s role in supporting outbreak response activities within their own countries [6].

In late 2006, Chretien et al., provided a detailed breakdown of how the broad-ranging DoD’s global disease surveillance network could potentially serve as a model for other global public health entities to adequately identify and respond to these complex threats [7]. This paper describes how the AFHSC-GEIS network has significantly contributed toward effective outbreak identification and response and capacity building within partner host countries [8] under the guiding principles of the WHO’s IHR (2005) [9]. For clarity and for the purposes of this assessment, we will describe the accomplishments of the AFHSC-GEIS network during fiscal year 2009 (Oct. 1, 2008 through Sept. 30, 2009) through two categories: respiratory disease outbreaks and non-respiratory EID outbreaks.

## Accomplishments

### General

**Figure 1:**
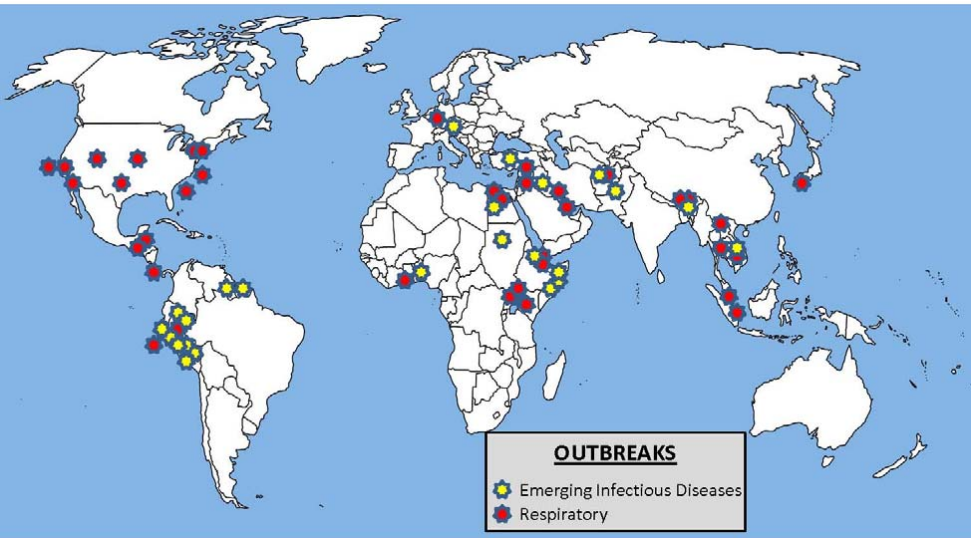
Global Snapshot of Emerging Infectious Diseases and Respiratory Diseases Outbreaks. October 2008 to September 2009

The AFHSC-GEIS network responded to 76 outbreaks (Table 1, Figure 1) in 53 countries during the 2009 fiscal year (FY09), several in direct support of the IHR (2005). The most common diseases investigated were influenza (47), cholera (four), dengue fever (four) and hepatitis (three). Human disease was present in all but one of these outbreaks, and specific causative agents were identified in 69 (92 percent) of them. The population affected ranged from less than 10 individuals to several thousand, and support efforts were often ongoing engagements beyond the initial investigation. The type of population supported also varied, depending on the relationship and the nature of the mission of the laboratory partner. Thirty-six (48 percent) of the outbreak investigations involved partners supporting civilian entities through formal bilateral requests or as part of their role as a WHO regional reference laboratory (NAMRU-3, AFRIMS and USAMRU-K). In the majority of these instances, testing of samples from civilian populations was performed. Twenty-four (32 percent) of the partner responses involved outbreaks among U.S. troops stationed in the continental United States (CONUS) or at overseas locations, while 15 (20 percent) of the responses involved investigations in collaboration with foreign military partners and multinational forces involved in peacekeeping activities or exercises. One investigation involved influenza testing of U.S. expatriates through the U.S. Embassy clinic in Jakarta, Indonesia.

**Table 1:** Diseases and agents investigated throughout 2009 among AFHSC-GEIS global partner laboratories and institutions.

Disease or Agent	Number of Outbreaks
Adenovirus	1
Campylobacter	1
Chikungunya	1
Cholera	4
Cyclospora	1
Dengue fever	4
Hepatitis (viral)	3
Influenza	
Pandemic (2009) H1N1	42
Seasonal Influenza	4
Avian Influenza (H5N1)	2
Malaria	1
Norovirus	1
Rickettsiosis	1
Rift Valley fever	1
Salmonella typhi	1
Group A Streptococcal (GAS) pneumonia	1
Syphilis	1
Unknown etiology	
Conjunctivitis	1
Gastrointestinal syndrome	2
Respiratory syndrome	1
Hemorrhagic syndrome	1
Vampire bat bites	1

Total	76

Response activities included a range of efforts from the provision of simple consultative services to comprehensive outbreak packages that included field support, epidemiologic consultation and laboratory diagnostic support. In 27 (36 percent) of the outbreaks, personnel were provided for field support, 44 (59 percent) outbreaks received epidemiologic or clinical consultative support, and laboratory diagnostic and testing support was provided to 68 (91 percent) of the outbreak support requests. Many AFHSC-GEIS partner responses were a combination of the above and 27 (36 percent) of the outbreak investigations provided a fully comprehensive response effort with support from all three categories.

### Respiratory disease outbreaks

Beginning in 2006, the DoD’s global disease surveillance network has worked to enhance the existing surveillance infrastructure to prepare for a potential influenza pandemic. The goals of these expansion efforts included broadening the network to monitor and detect increasing numbers of avian (H5N1) influenza outbreaks around the world and identify new infectious disease threats [10]. This expansion of capacity and function was both appropriate and fortuitous as AFHSC-GEIS network partners at NHRC and USAFSAM were the first in the world to detect the novel influenza A/H1N1 strain in April 2009 in San Diego, California, and San Antonio, Texas. This rapid detection during the end of the influenza season allowed the appearance of this novel strain to be identified and reported as a Public Health Emergency of International Concern by the CDC, a WHO Collaborating Center, in compliance with IHR (2005). With the onset of this influenza A/H1N1 pandemic in April 2009, substantial efforts were made by AFHSC-GEIS network partners to assist the global health community in responding to this threat. Fifty-one (67 percent) of the 76 outbreaks responses involved respiratory diseases, 41 (80 percent) of which were due to novel influenza A/H1N1.

Beginning in April 2009, with the onset of the influenza pandemic, disease surveillance and investigative support activities were dominated by novel influenza A/H1N1-related responses. As with other investigations, the activities for novel influenza A/H1N1 were wide-ranging and involved different populations and situations. The AFHSC-GEIS network supported the diagnostic confirmation (directly in DoD lab or through support of host-country laboratories) of the first cases in 14 countries (Bhutan, Cambodia, Colombia, Djibouti, Ecuador, Egypt, Kenya, Kuwait, Lao People’s Democratic Republic, Lebanon, Nepal, Peru, Republic of the Seychelles and the United States), again demonstrating direct support for increasing compliance with IHR (2005). The non-U.S. activities were a result of the respective AFHSC-GEIS partner laboratory’s roles as regional reference testing centers and the bilateral collaborations with host-country Ministries of Health. These bilateral relationships resulted in support of 17 large-scale outbreaks among civilians in 13 countries.

U.S. service members and beneficiaries were affected by the pandemic from the beginning. In the first wave (April through August 2009), AFHSC-GEIS network partners actively investigated 18 different outbreaks on U.S. military installations and among previously defined high-risk groups [11]. These high-risk groups included deployed or deploying personnel, shipboard personnel, new accessions (basic and advanced military trainees and service academy students), health care workers, children and staff in daycare centers, and pregnant women. Stressful military environments, highly mobile missions and complex troop dynamics helped to propagate pandemics in the past and have drawn the attention of the military’s operational leadership and leaders of civilian sectors within host countries. These investigations involved from a few dozen cases to more than 1,000 cases tested.

Shipboard investigations involved a number of responding units and included ships at sea in the Atlantic and Pacific Oceans, and the Persian Gulf, as well as vessels in port in major cities around the world. Shipboard investigations have benefitted from the ongoing surveillance of respiratory disease on large U.S. Navy ships [12]. Patients meeting the case definition of febrile respiratory illness (FRI), temperature > 100.4°F and cough or sore throat, undergo throat swabs and the specimens are stored in liquid nitrogen or -80°C freezers. This surveillance, conducted by NHRC, has facilitated timely specimen collection and pathogen identification in a number of shipboard respiratory outbreaks in recent years. The surveillance system detects a wide variety of circulating influenza types as these ships make numerous worldwide port stops over a short time period. In general, the specimens are saved and processed at the end of a ship’s deployment, which usually lasts six months. However, in the case of an outbreak, febrile respiratory illness (FRI) specimens from immediately before the outbreak onward can be processed onboard the ship with specific polymerase chain reaction (PCR) testing or shipped overnight to NHRC for 12- to 24-hour turnaround molecular testing and culture.

Shipboard investigations ranged from simple identification of the novel influenza A/H1N1 virus to detailed epidemiologic investigation and testing [13]. NMRCD, in partnership with the Peruvian Navy, investigated a shipboard outbreak in June through July 2009 aboard a 355-crew Peruvian Navy ship at sea in the Pacific Ocean. During the four-week investigation, team members confirmed 78 of 85 (92 percent) febrile acute respiratory illness cases as novel influenza A/H1N1. The attack rate aboard the ship during the time of the outbreak was 22 percent. Early detection, through an active shipboard surveillance system modeled on the NHRC program, played an important role in the rapid detection and subsequent control of the outbreak [14].

**Figure 2:**
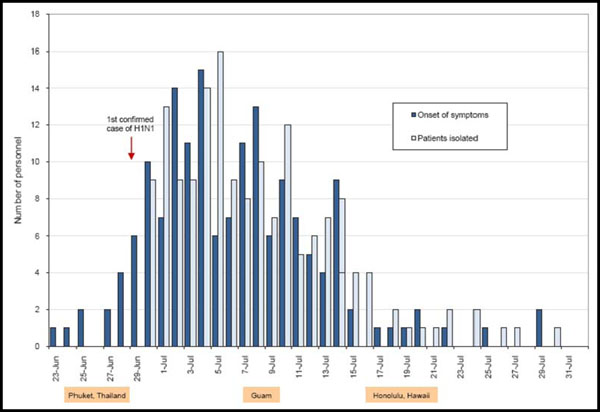
Epi curve of cases, isolated patients during A/H1N1 outbreak aboard the USS Boxer. Summer 2009.

The USS Boxer (LHD 4), with a complement of more than 2,200 U.S. Sailors and Marines, was docked at Phuket, Thailand, from June 23 through 29, 2009. On June 30, four of 14 patients with ILI tested positive for influenza A by PCR. By July 9, 102 individuals had provided respiratory specimens (throat swabs in viral transport media) that were sent to NHRC, where 69 were confirmed as novel influenza A/H1N1. Overall, more than 200 cases were identified in a five-week period and 177 personnel were isolated with FRI for an average duration of 3.6 days (Figure 2) [15].

As part of the deployment process, troops usually transition through pre-deployment training at what the U.S. Army refers to as power projection platforms (PPP). These are usually large installations with thousands of individuals transferring to the deployed setting each year [16]. The first wave of novel influenza A/H1N1 included outbreaks among deploying service members from nine of the 15 PPPs and subsequently resulted in two large-scale outbreaks in the operational theatre (Iraq and Kuwait). The most notable PPP outbreaks were at Fort Riley, Kansas (n=33), Fort Hood, Texas (n=44), Fort Lewis, Washington (n=144), and Fort Bliss, Texas (n=188). Response activities at each of the PPPs varied based on the reality on the ground and timing in the pre-deployment process. Sites with large numbers of individuals in the latter stages of pre-deployment were forced to take much more aggressive steps to screen and monitor the symptoms of illness for departing troops.

**Figure 3:**
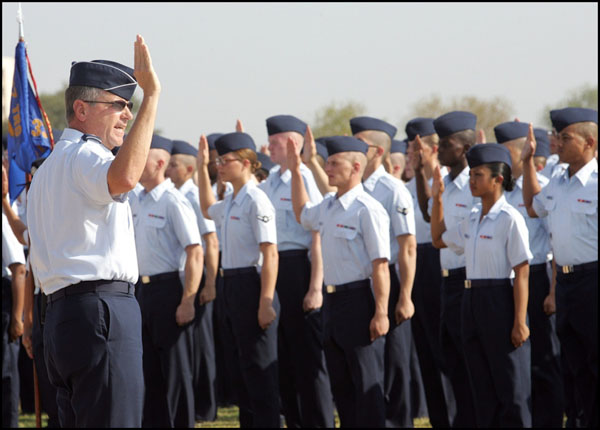
U.S. Air Force Academy swearing-in ceremony (Photo Courtesy of Official U.S. Air Force Academy website)

**Figure 4:**
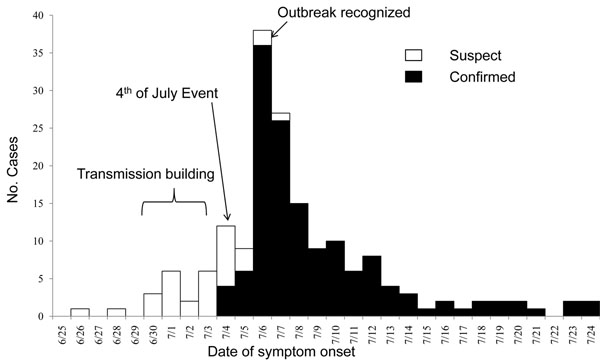
Confirmed, suspect cases of A/H1N1 virus infection at U.S. Air Force Academy, 2009 [17]

Military service academies were particularly hard hit by novel A/H1N1 outbreaks during the first wave. Notable outbreaks requiring outside assistance and support took place at the U.S. Air Force Academy (USAFA) [17] (Figure 3), U.S. Coast Guard Academy, U.S. Military Academy and U.S. Naval Academy. Response efforts ranged from diagnostic support for cadets and midshipmen in isolation to comprehensive outbreak support. The response effort for USAFA was unique because it was the first to provide information on the different aspects of the disease in such a high-risk setting. The USAFA investigation team conducted retrospective and prospective surveillance to describe the epidemiology of the outbreak and to define and implement effective control measures. Extensive education and hygiene efforts were implemented and ill cadets were isolated from non-ill cadets to decrease transmission. The team documented confirmed (n=134) and suspected (n=34) novel influenza A/H1N1 cases among basic cadet trainees, with an outbreak period incidence rate of 11 percent. The peak of the outbreak occurred on July 6 and was likely propagated by a Fourth of July social-mixing event (Figure 4). The investigation team obtained serial nasal wash samples from patients and published the first report of virus shedding duration determined by virus culture. Follow-up nasal wash samples taken seven days from illness onset and from asymptomatic patients (≥24 hours) contained cultureable virus in 24 percent and 19 percent of the samples, respectively. The USAFA investigation made a significant and novel contribution to the general public health knowledge on the transmission dynamics of this disease and demonstrated a high proportion of asymptomatic, sub-clinical disease among cadets in this setting.

**Figure 5:**
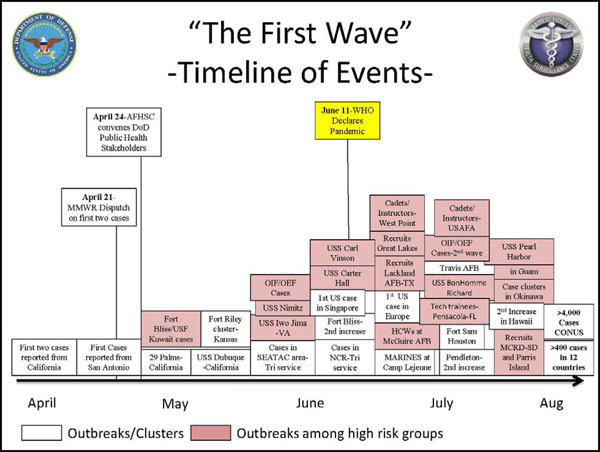
A/H1N1 epi curve, timeline for outbreaks and clusters among U.S. service members and beneficiaries.

Remarkably, while notable novel influenza A/H1N1-related outbreaks occurred at all of the basic military training centers, overall, military recruits were only minimally affected during the first wave of the pandemic (Figure 5). Subsequently, the burden of disease shifted almost entirely to recruits at these installations in early fall (August-October) 2009, although the disease burden of novel influenza A/H1N1 among recruits during the entire time was significantly smaller than the ongoing epidemic of adenovirus respiratory diseases among U.S. military recruits [18].

In addition to the novel influenza A/H1N1 outbreaks, four seasonal influenza outbreak investigations were undertaken: one among deployed U.S. troops in Djibouti, one in a refugee camp in Nepal, one onboard a U.S. Navy submarine, and one among newly arrived Japanese military trainees in Camp Pendleton, California. Two H5N1-related investigations occurred, along with an ongoing investigation of human H5N1 cases in Egypt and assistance with human testing during an outbreak of influenza in birds in Nepal in November of 2008. Additionally, two respiratory illness outbreak investigations occurred among basic military trainees, while the U.S. Army Public Health Command (Provisional) investigated an outbreak of Group A Streptococcus at Fort Leonard Wood, Missouri. An adenovirus type B14 was investigated at the U.S. Coast Guard Training Center in Cape May, N.J.. Both outbreaks took place in March 2009 and were supported by the laboratory at NHRC.

Overall, the AFHSC-GEIS global disease surveillance network response remained strong through the end of the first wave and well into the second fall wave of the pandemic. At that point, efforts across the network primarily shifted from outbreak response to systematic surveillance (monitoring and representative sampling) of affected sites, along with more complex characterization of the viruses and close tracking of the global circulation patterns of all influenza viruses.

### Non-respiratory, emerging infectious disease outbreaks

The AFHSC-GEIS EID surveillance program includes five program pillars or disease focus areas: respiratory diseases, febrile and vector-borne illnesses, enteric diseases, sexually transmitted infections and antimicrobial resistant organisms. Outbreaks involving all five pathogen/illness categories occurred in the one-year timeframe of this report. Global surveillance network partners responded to 20 outbreaks involving non-respiratory EIDs where the disease agents were identified, including four dengue fever, four cholera, three viral hepatitis, two gastrointestinal syndrome, and one each of *Campylobacter*, chikungunya, *Cyclospora*, malaria, Norovirus, rickettsiosis, Rift Valley fever, *Salmonella typhi* and syphilis. Additionally, partners conducted individual outbreak investigations of conjunctivitis and viral hemorrhagic syndrome, while one response activity examined bites from vampire bats. Overall, these response activities took place in 14 countries.

From May to September 2009, a diarrheal disease outbreak occurred in western Nepal, with reports of nearly 70,000 patients treated and over 350 deaths. The National Public Health Laboratory of the Ministry of Health and Population for Nepal requested assistance from the Walter Reed/AFRIMS Research Unit Nepal (WARUN) in determining the etiology. WARUN received 158 stool samples from eight districts in western Nepal; 45 percent from children <15 years and 61 percent from female patients. WARUN in Nepal and AFRIMS in Thailand analyzed the samples using real-time PCR and culture. Eighty-two of the outbreak samples were positive for *Vibrio cholerae* (01/0139) by PCR. Many of the PCR positive samples subsequently grew *V. cholerae* serogroup 01 serotype Ogawa in culture. Antibiotic sensitivity analysis of the *V. cholera* isolates showed universal resistance to nalidixic acid, colistin, streptomycin, sulfisoxazole, and trimethoprim/sulfamethoxazole but sensitivity to the commonly recommended antibiotics azithromycin, ciprofloxacin, ampicillin and tetracycline. The National Public Health Laboratory performed the initial culture on these stool samples, and the WARUN and AFRIMS laboratories eventually verified and confirmed the cause of the outbreak. The effort enabled local health authorities to institute appropriate treatment and prevention measures in a timely manner.

Department of Defense resources have also been directed toward the investigation or characterization of other phenomena that may threaten public health other than the disease incidence. For example, AFRIMS and NAMRU-2 investigators were among the first worldwide to recognize that increases in *Plasmodium falciparum* treatment failures to the powerful artemisinin combination therapies signaled the onset of resistance to the last remaining class of malaria treatments in widespread deployment today. As a result of these findings, worldwide efforts are under way to contain spread of drug-resistant forms of the parasite from what is hoped to be a relatively constrained area in Southeast Asia along the Thai-Cambodian border [19].

Although many methods may be used to detect outbreaks, most often an astute clinician or laboratory worker identifies an unusual occurrence or an increased frequency. However, syndromic or electronic surveillance may serve as another method to augment, but not replace, traditional disease surveillance efforts [8]. AFHSC-GEIS partners at NMRCD have implemented a near real-time electronic disease surveillance system based on highly cost-effective and sustainable strategies affordable in resource-constrained, developing countries. This system is called Alerta and was first implemented in the Peruvian Navy population in 2002, the Peruvian Army three years later and more recently the Peruvian Air Force. Currently, Alerta covers 98 percent, 99 percent and 95 percent of the Peruvian Navy, Army and Air Force population, respectively, and receives over 200 notifications per week. The system has been useful in outbreak detection, identifying over 20 outbreaks in the last year, six of which were for influenza. Eighty-six reporting units operate nationwide, including border units, hospitals, infirmaries and ships incorporated in the Peruvian Navy surveillance system. Due to this previous experience, the Peruvian Army's expansion has been faster, currently receiving reports from 120 units throughout the country.

Within the one-year period of review, the 76 outbreaks identified by the AFHSC-GEIS network spanned the clinical disease spectrum and involved efforts of international significance. The U.S. DoD has established a robust and flexible global response framework through open collaborations with long-standing host country partners, other global health institutions and agencies such as the WHO, the CDC and Institute Pasteur. In doing so, AFHSC-GEIS, through its global network of partners, is addressing the specific milestones for building sustained capacity for early detection and rapid response as prescribed by the IHR (2005).

## Conclusions

The Armed Forces Health Surveillance Center’s Division of Global Emerging Infections Surveillance and Response System continues to expand internationally to effectively identify and respond to threats from a wide range of disease agents and geography. The growth and enhancement of this surveillance system in anticipation of pandemic/avian influenza allowed the DoD to identify the current influenza pandemic [20,21] and a number of other infectious disease outbreaks in communities throughout the globe. A multipurpose system with defined goals and pillars of focus, the AFHSC-GEIS network has evolved to become a true model for emerging infectious surveillance platforms at the local, regional and international level. By utilizing this established global system, the DoD is able to provide a common and systematic approach to disease surveillance and a framework for effective response. As emerging and re-emerging threats develop in areas where partners work collaboratively with host countries and global health institutions, the AFHSC-GEIS network stands ready to respond.

## Competing interests

To the best knowledge of the authors, there are no competing interests.
